# Determinants of hyperphosphatemia and its relation with hemoglobin levels among hemodialysis patients in two hospitals from Syria: a prospective cross-sectional study

**DOI:** 10.1097/MS9.0000000000000632

**Published:** 2023-04-14

**Authors:** Mohammad Alsultan, Marwa Kliea, Mohamed T. Anan, Baraa Abdulkader, Reem Kazkaz, Abdullah Al Sultan, Mohamad Al Masri, Qussai Hassan, Kassem Basha

**Affiliations:** a Department of Nephrology; b Department of Neurology; c Department of Orthopedic Surgery, Al Assad and Al Mouwasat University Hospitals, Damascus University, Faculty of Medicine, Damascus; d Department of Nephrology, Al Assad University Hospital; e Department of Nephrology, Al Mouwasat University Hospital, Damascus University, Faculty of Medicine, Damascus; f Department of Statics, Faculty of Sciences, Aleppo University, Aleppo

**Keywords:** anemia, hemodialysis (HD), hemoglobin (Hb), hyperphosphatemia, phosphorus (phos), Syria

## Abstract

Hyperphosphatemia and anemia were both associated with several complications in chronic kidney disease (CKD) patients. This study aimed to determine the risk factors of hyperphosphatemia and its relation with anemia among hemodialysis (HD) patients. Secondly, it aimed to determine the prevalence of hyperphosphatemia and anemia.

**Material and methods:**

A prospective cross-sectional study was conducted among 146 HD patients from two HD centers in Syria, between June 2021 and March 2022. All patients at least 18 years old on maintenance HD were enrolled. The threshold of phosphorus (phos) level was divided by the upper normal range among HD patients (5.5 mg/dl). We used parametric and nonparametric statistics, the Pearson and Spearman correlations with simple and multiple linear regressions between study variables.

**Results:**

36.9% of patients had a serum phos level of 5.5 or less (norm phos group), and 63.1% of patients had a serum phos level higher than 5.5 (high phos group). Also, 60.9% of patients had hemoglobin (Hb) less than 10 g/dl, and 40.4% of patients had Hb at least 10 g/dl. Age, type of HD access, phos binders (P-binders), parathyroid hormone (PTH), and calcium (Ca) showed significant effects on phos levels. Most patients were using arteriovenous fistula (AVF) (89.7%) as a HD access, and the meantime on HD was higher in the norm phos group compared to the high phos group. In a multivariate and univariate logistic regression analysis, hyperphosphatemia increased with increasing urea (Ur) and creatinine (Cr) levels, while the odds declined with increasing time on HD. Hb did not show a significant relation with phos by using several statistical methods.

**Discussion/Conclusion:**

A high prevalence of hyperphosphatemia and anemia was encountered among this sample of HD patients from Syria. There was no correlation between phos and Hb levels in contrast to previous conflicting studies, which mandates future studies to evaluate this correlation and further efforts to determine the range of phos that could have a benefit on anemia with respect to other comorbidities.

## Introduction

Highlights

- Hyperphosphatemia and anemia was reported in 63.1 and 60.9%, respectively.
- Age, type of hemodialysis (HD) access, phos binders (P-binders), parathyroid hormone (PTH), and calcium (Ca) have effects on phosphorus (phos) levels.
- The meantime on HD had an inverse effect on phos.
- Hyperphosphatemia increased with increasing urea (Ur) and creatinine (Cr) levels.
- Hemoglobin (Hb) did not show a significant relation with phos by using several statistical methods.

Phosphorus (phos) is an important element involved in various vital functions including signal transduction, cell membrane function, energy exchange, and bone mineral metabolism^1^. Hyperphosphatemia in chronic kidney disease (CKD) patients is explained as a reduction in the ability of kidneys to remove phos as phos reabsorption is maximally inhibited and renal excretion cannot increase once estimated glomerular filtration rate (eGFR) falls below 30 ml/min/1.73 m^2^. At this point, serum phos and parathyroid hormone (PTH) must increase^1,2^. Approximately 15% of CKD stage 4 patients will have hyperphosphatemia and it increases to 50% in those with CKD stage 5 predialysis^1^.

Hyperphosphatemia, which is a component of mineral bone disease (MBD), is associated with rapid progression of established CKD, and even phos levels in the high normal range (≥4 mg/dl and <4.5 mg/dl) are found to be associated with increased risk for CKD^3–5^. Several observational studies demonstrate that elevated serum phos is an independent risk factor for cardiovascular disease (CVD), and vascular calcification in CKD patients with vascular mortality is nearly 50%^6–10^. Furthermore, elevated phos is linked to high rates of mortality in CKD patients, in both dialysis and non-dialysis patients^3,6,7,11^. This was best illustrated in a meta-analysis of 13 studies that included 92 345 patients with CKD, over 97% of whom were on dialysis; the risk of death increased by 18% for every 1-mg/dl increase in serum phos^12^. Due to the close relationship with PTH, it may also be speculated that PTH’s effect on mortality may also be indirectly reflecting the effect of hyperphosphatemia^10^.

Anemia is also associated with increased morbidity and mortality related to CVD and an increased risk of hospitalization and hospital stay^13,14^. Anemia accompanies abnormal mineral metabolism during progressive renal failure, largely due to erythropoietin deficiency, iron (Fe) deficiency, inflammation, oxidative stress, bone marrow fibrosis, blood loss, malnutrition, and shortened red blood cell survival. Several investigators have suggested a link between anemia and MBD^15–17^.

Hyperphosphatemia was associated with anemia independent of other MBD components that were observed in a few studies in non-dialysis CKD and kidney transplant patients^18,19^. As hemodialysis (HD) patients represent a different population, this association between hyperphosphatemia and anemia is much less well-studied and it remains a controversial issue. Meanwhile, hyperphosphatemia and anemia were both associated with several complications in HD patients and there were no previous studies from our country in this regard. The objective of our study is to determine the risk factors of hyperphosphatemia among HD patients and to also evaluate its relation with anemia. The secondary aim is also to determine the prevalence of hyperphosphatemia and anemia among HD patients.

## Material and methods

A prospective cross-sectional study was conducted among HD patients from two HD centers from Syria, between June 2021 and March 2022. The study protocol was approved by the Research Ethics Committee of Damascus University and in accordance with the Declaration of Helsinki and in line with the STROCSS (strengthening the reporting of cohort, cross-sectional and case–control studies in surgery) guidelines^20^.

All patients at least 18 years old on maintenance HD were enrolled. Exclusion criteria were: refused to participate and missing data of phos or hemoglobin (Hb). All data, which included clinical and laboratory information, were collected by three nephrology residents. Of a total of 209 patients, 65 patients were excluded. Of them, two patients were below 18 years, 40 patients refused drawing tests, and 23 patients refused participation in the study. Data from participants included the following: age, gender, BMI, smoke (pack years), type of HD access, and time on HD (months). Medical history included existing hepatitis B virus (HBV) or hepatitis C virus (HCV) infection, history of diabetes mellitus (DM), hypertension (HTN), CVD, kidney transplantation (KT), and history of pulmonary, neurologic, and other disorders. Additionally, current drugs were inserted as angiotensin-converting enzyme inhibitors and angiotensin-receptor blockers (ACEIs/ARBs), calcium channel blockers (CCBs), diuretics, alpha-blockers, antiplatelet (anti-PLT), statins, epoetin alfa (EPO), Fe supplements, calcium (Ca) supplements, phos binders (P-binders), uric acid lowering therapy (ULT), and alfa calcitriol. Laboratory tests included white blood cells (WBCs), Hb, hematocrit (HT), platelets (PLTs) count, urea (Ur), creatinine (Cr), sodium (Na), potassium (K), Ca, Fe, albumin (ALB), phos, and PTH. All laboratory tests were conducted every 3–6 months, before the programmed HD session and were conducted using the same laboratory in each hospital.

Detailed patient data were collected at the study entry (baseline) in each facility. Hb and phos were collected on two occasions apart; in the beginning of data collection and after 4 months, to be enrolled in statistical analysis. A general practice for all patients mandates drug adjustments in intervals depending on laboratory tests or the clinical status. Patients were given subcutaneous EPO (4000 IU per ampule) that protocol was based on Hb levels, with target Hb levels between 10 and 11.5 g/dl^21^. EPO was administered as the number of ampules per week except who received at least three ampules, which was inserted as three. The type of HD access was inserted as non-cuffed (temporary), cuffed (permanent) catheter, and arteriovenous fistula (AVF). Most patients were receiving maintenance Fe supplements either orally or intravenously. BMI was calculated by dividing weight (in kilograms) by height (in meters squared).

As the study was conducted in HD patients, the threshold of phos level was divided by the upper normal range among HD patients (5.5 mg/dl)^22^.

### Statistical analysis

Statistical analysis was performed using the program R 4.02 and SPSS version 23.0. We used nonparametric statistics such as the *χ* ^2^ test and parametric statistics such as analysis of variance (ANOVA). The measure of correlation was conducted using the Pearson and Spearman correlation coefficients. Descriptive analysis was performed using mean and standard deviation (mean±SD). Also, we used simple and multiple linear regressions between study variables. *P* value less than 0.05 was considered statistically significant.

## Results

Of the 146 HD patients that were enrolled in this prospective study, 54 patients (36.9%) had a serum phos level of 5.5 mg/dl or less (norm phos group) and 92 patients (63.1%) had a serum phos level of more than 5.5 mg/dl (high phos group). Also, 87 patients (60.9%) had Hb less than 10 g/dl, and 59 patients (40.4%) had Hb at least 10 g/dl. Baseline demographic and laboratory characteristics are shown in Tables 1 and 2, respectively.

**Table 1:** Baseline demographic characteristics of patients grouped according to serum phosphorus level

Variables	*P*≤5.5 (*N*=54, 36.9%)	*P*>5.5 (*N*=92, 63.1%)	*P*
Age (mean±SD)	49.11±16	43.27±14	0.02
Gender (*N*/%)	F (23/ 15.75%)	F (37/ 25.34% )	0.09
	M (31/ 21.23%)	M (55/ 37.67%)	
Smoke (pack years)	10.7±1.8	10.02±18.4	0.82
BMI (mean±SD)	21.9±5.3	23.2±5.3	0.52
HD access			
Temporary (N)	4	4	0.05
Permanent (*N*)	3	4	
Fistula (*N*)	47	84	
HD time (mean±SD) (months)	72.62±68.7	59.6±58.9	0.23
Medical history			
HBV (*N*)	–	3	0.36
HCV (*N*)	4	10	0.31
HTN (*N*)	45	77	0.82
CVD (*N*)	17	22	0.24
DM (*N*)	3	4	0.12
Transplant (*N*)	6	8	0.12
Pulmonary (*N*)	5	1	0.10
Neurologic (*N*)	1	6	0.86
Others (*N*)	14	18	0.60
Drugs			
ACEI/ARBs (*N*)	5	15	0.17
CCB (*N*)	32	65	0.23
BB (*N*)	22	47	0.30
Diuretics (*N*)	16	19	0.89
α Blockers (*N*)	14	33	0.14
Anti-PLT (*N*)	16	16	0.08
Statins (*N*)	9	8	0.37
EPO (*N*)			
1 (4000 IU/W)	12	10	0.12
2 (8000 IU/W)	30	56	
≥3 (≥12 000 IU/W)	9	21	
Iron (*N*)	41	73	0.97
Calcium sup (*N*)	47	72	0.17
P-binders (*N*)	11	26	0.01
ULT (*N*)	14	19	0.37
α Calcitriol (*N*)	14	36	0.22

ACEIs, angiotensin-converting enzyme inhibitors; ARBs, angiotensin-receptor blockers; BB, β blocker; CCB, calcium channel blocker; CVD, cardiovascular disease; DM, diabetes mellitus; EPO, epoetin alfa; F, female; HBV, hepatitis B virus; HCV, hepatitis C virus; HD, hemodialysis; HTN, hypertension; M, male; PLT, platelet; ULT, uric acid lowering therapy.

**Table 2:** Baseline laboratory characteristics of patients grouped according to serum phosphorus level

	Phos		
Lab variables	≤5.5	>5.5	*P*
WBC	6.32±2.6	6.36±2.29	0.92
Hb	9.5±2.08	9.3±1.9	0.45
HT	29.4±5.6	28.7±5.02	0.50
PLT	218.7±68.02	200.11±84.2	0.17
Ur	127.4±39	167.1±42.9	0.001
Cr	9.8±3.3	12.5±3.09	0.001
Na	138±4.9	138.2±4.2	0.76
K	5.2±0.7	5.5±0.81	0.02
Ca	8.08±1.15	8.12±1.1	0.82
Fe	52.4±30.8	48.07±25.5	0.36
ALB	3.8±0.4	3.9±0.4	0.17
PTH	781.3±712.3	114.5±1069	0.03

ALB, albumin; Ca, calcium; Cr, creatinine; Fe, iron; Hb, hemoglobin; HT, hematocrit ; K, potassium; Na, sodium; Phos, phosphorus, PLT, platelet; PTH, parathyroid hormone; Ur, urea; WBC, white blood cells.

From Table 1, age, type of HD access, and P-binders showed statistically significant effects between phos groups (0.02, 0.05, and 0.01, respectively). The mean age was (49.11±16) in the norm phos group and (43.27±14) in the high phos group. The norm phos group included 21.23% males and 15.75% females compared to the higher number of males and females in high phos group (25.34 and 37.67%, respectively). Most patients were using AVF (*n*=131, 89.7%) as a HD access. The meantime on HD was higher in the norm phos group (72.62±68.7 months) compared to the high phos group (59.6±58.9 months) (*P*=0.05). The most common comorbidity was HTN (*n*=122, 83.5%) followed by CVD (*n*=39, 26.7%). The most common anti-HTN drug used was CCB (*n*=97, 66.4%), followed by β blockers (BB) (*n*=69, 47.2%) and α blockers (*n*=47, 32.1%). Most patients received EPO (*n*=138, 94.5%) but the dose did not show a significant effect between phos groups (*P*=0.12). In addition, the majority received Fe and Ca supplements (*n*=114, 78% and *n*=119, 81.5%, respectively). Only a quarter of patients (*n*=37, 25.3%) received P-binders other than Ca-containing binders, which showed a statistically significant difference (*P*=0.01).

Based on baseline laboratory tests (Table 2), the mean serum levels of Ur and Cr were higher in high phos group (167.1±42.9 and 12.5±3.09, respectively) versus the norm phos group (127.4±39 and 9.8±3.3, respectively), and showed statistically significant differences (*P*=0.001, for each). In the same regard, K showed statistically significant difference (*P*=0.02) and was higher in the high phos group (5.5±0.81) compared to the norm phos group (5.2±0.7). PTH showed a statistically significant effect on phos level (0.03), and higher levels of PTH were seen in the norm phos group (781.3±712.3) compared to the high phos group (114.5±1069). The mean Hb level was nearly comparable between the norm phos group (9.5±2.08) and the high phos group (9.3±1.9) but did not show statistically significant difference (*P*=0.45), hence both consists with severe anemia^21^.

In a multivariate logistic regression analysis (Table 3), the odds of having hyperphosphatemia increased with increasing serum Ur levels, which showed a positive influence [odds ratio (OR) 1.027, 95% CI, *P*=0.002]. The odds of having hyperphosphatemia declined with increasing HD time, which showed a protective influence (OR 0.988, 95% CI, *P*=0.019). Hb did not display statistically significant effect on phos levels (−0.154, OR=0.857, *P*=0.62), as so for the remaining variables.

**Table 3:** Results of multivariate logistic regression model of phosphorus with 95% CI, odds ratio for having phosphorus concentration ≤5.5 versus >5.5 mg/dl by numerical baseline patient measures

Variable	Coefficient	Odds ratio	*P*
BMI	−0.013	0.987	0.79
HD time	−0.012	0.988	0.019
WBC	0.022	1.022	0.83
Hb	−0.154	0.857	0.62
HTN	0.003	1.003	0.97
PLT	0.0008	1	0.83
Ur	0.027	1.027	0.002
Cr	0.121	1.128	0.32
Na	0.026	1.026	0.68
K	0.238	1.268	0.56
Ca	0.249	1.282	0.38
Fe	−0.0005	0.999	0.95
PTH	0.0002	0.999	0.47

ALB, albumin; Ca, calcium; Cr, creatinine; Fe, iron; Hb, hemoglobin; HD, hemodialysis; HTN, hypertension; K, potassium; Na, sodium; PLT, platelet; PTH, parathyroid hormone; Ur, urea; WBC, white blood cells.

The relationship between phos concentrations and every variable solely was analyzed by using univariate logistic regression analysis (Table 4). The odds of having hyperphosphatemia increased with increasing serum Ur levels, which showed a positive impact (OR 1.026, 95% CI, *P*=0.0001). Also, Cr level showed a statistically significant impact (*P*=0.003) on phos concentrations, where the odds of having hyperphosphatemia increased with increasing Cr levels (OR 1.279, 95% CI). Hb did not display statistically significant effect on phos levels (−0.019, OR=0.98, *P*=0.86), as so for the remaining variables. Also, studying the linear regression of phos level independently with both HD time and Ur showed statistically significant relations (*P*=0.019 and <0.0001, respectively). HD time was protective against hyperphosphatemia (−0.010, OR=0.989) and Ur showed a positive impact (0.031, OR=1.032).

**Table 4:** Results of univariate logistic regression model of phosphorus with 95% confidence interval, odds ratio for having phosphorus concentration ≤5.5 versus >5.5 mg/dl by numerical baseline patient measures

Variable	Coefficient	Odds ratio	*P*
BMI	−0.028	0.971	0.454
HD time	−0.004	0.995	0.17
WBC	0.009	1.009	0.90
Hb	−0.019	0.980	0.86
HTN	−0.0003	0.999	0.99
PLT	−0.001	0.998	0.64
Ur	0.026	1.026	0.0001
Cr	0.246	1.279	0.003
Na	0.013	1.013	0.78
K	0.561	1.753	0.07
Ca	0.066	1.068	0.74
Fe	−0.003	0.996	0.57
PTH	0.0002	1	0.29
The relation of phos with HD time and Ur			
** **HD time	−0.010	0.989	0.019
** **Ur	0.031	1.032	<0.0001

Ca, calcium; Cr, creatinine; Fe, iron; Hb, hemoglobin; HD, hemodialysis; HTN, hypertension; K, potassium; Na, sodium; PLT, platelet; PTH, parathyroid hormone; Ur, urea; WBC, white blood cells.

**Figure 1:**
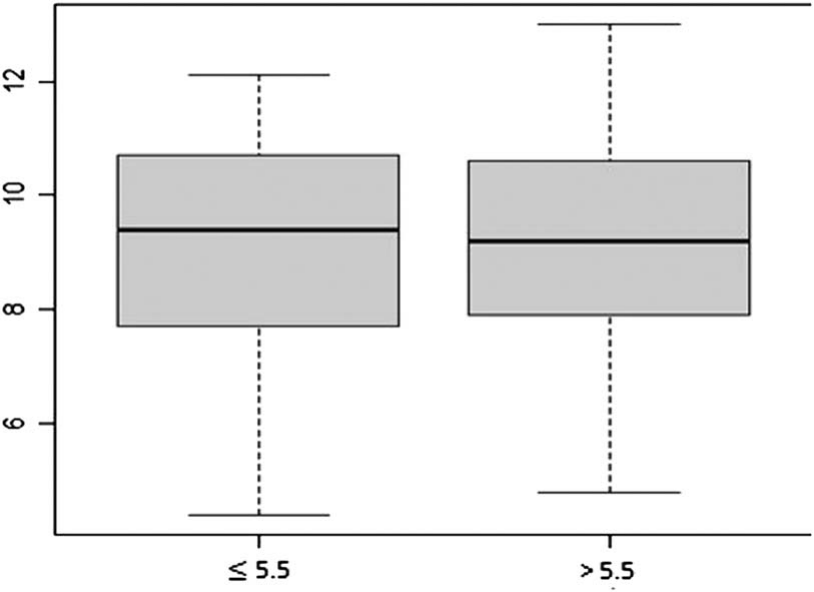
Boxplot: shows equal distributions of hemoglobin levels between phosphorus thresholds.

**Figure 2:**
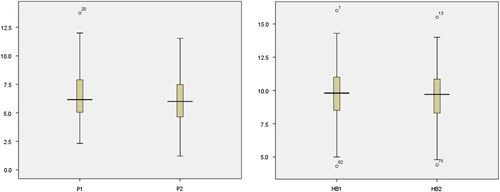
Boxplots: show similar distributions of phosphorus and hemoglobin levels between two measurements, 4 months apart. 1: referred to tests in the first month, at study entry. 2: referred to tests in the second month after 4 months.

**Figure 3:**
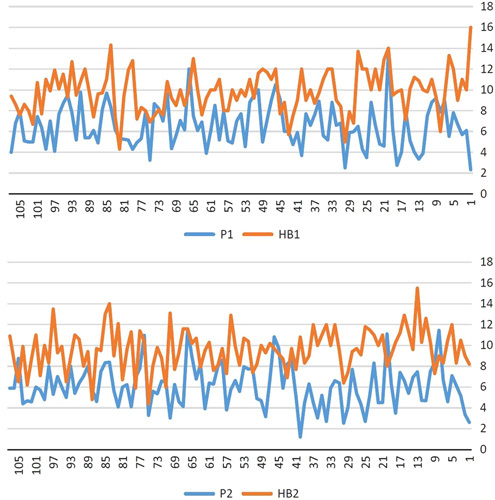
Distributions of hemoglobin and phosphorus levels between two cycles, 4 months apart. 1; Referred to tests in the first month, at study entry. 2: Referred to tests in the second month after 4 months.

Studying the relation between phos and Hb showed a weak correlation (*r*=0.029, OR=0.98) with no statistical significance (*P*=0.72). Hb levels were nearly equal with medians showing similar distributions between phos threshold (Fig. 1). Data were collected at study entry (baseline) and at 4 months thereafter. Regression analysis (Table 5) and boxplots of phos and Hb levels between these two measurements were built. There were no statistically significant relations between levels of phos and Hb (Table 5). Phos levels were nearly equal with medians showing similar distributions (Fig. 2). Also, Hb levels were nearly equal with medians showing similar distributions (Fig. 2). The interquartile range was comparable in phos and Hb levels between two cycles of measurements (Fig. 2). Distributions of Hb and phos levels between two cycles are shown in Figure 3.

**Table 5:** Regression analysis in two cycles of hemoglobin and phosphorus levels, 4 months apart

Variable (mean±SD)		*P*	
		Hb1^a^	Hb2^b^
		9.81±2.02	9.60±1.96
Phos 1^a^		0.42	0.43
6.51±2.06			
Phos 2^b^		0.62	0.98
6.10±2.02			

a 1: referred to tests in the first month, at study entry.

b 2: referred to tests in the second month after 4 months.

Phos, phosphorus.

**Figure 4:**
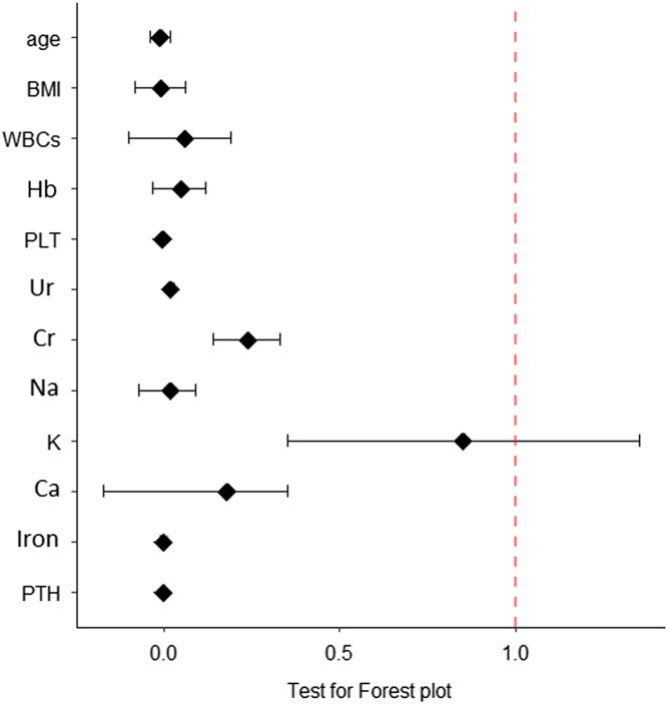
Forest plot: shows the odds ratio of hyperphosphatemia in relation to age, BMI, WBC, Hb, PLT, Ur, Cr, Na, K, Ca, iron, and PTH. *P* value is 0.05 or less. Ca, calcium; Cr, creatinine; Hb, hemoglobin; K, potassium; Na, sodium; PLT, platelet; PTH, parathyroid hormone; Ur, urea; WBC, white blood cells.

Forest plot analyses were performed to determine the relation of phos levels with remaining variables and we built models after being statistically significant (*P*≤0.05) (Fig. 4). Ca and Cr had evident effects on phos levels, while all remaining variables close to zero, including Hb, did not reveal an impact on phos levels.

## Discussion

To the best of our knowledge, this is the first study that evaluates the prevalence and risk factors of hyperphosphatemia among HD patients from Syria, in addition to studying its correlation with Hb levels. The prevalence of hyperphosphatemia was 63.1%. In demographic characteristics, age, type of HD access, and P-binders showed a statistically significant effect on phos levels. Also, by laboratory characteristics, Ur, Cr, and PTH showed a statistically significant impact on phos levels. The odds of having hyperphosphatemia increased with increasing serum Ur and Cr levels, while the odds declined with increasing time on HD. Also, Ca had an evident effect on phos levels only by forest plot. Hb and phos did not display a statistically significant relation by using different statistical analyses.

Of the 1005 HD patients studied from 38 dialysis centers in Egypt, hyperphosphatemia was present in 69.1% of cases, and among the 80 HD patients studied in the United Arab Emirates, 59 (73.8%) presented with hyperphosphatemia^23,24^. Also, only 44% of the patients in the Dialysis Outcomes and Practice Patterns Study (DOPPS II) achieved serum phos within the recommended range^25^. These results were comparable to our study, which showed the prevalence of hyperphosphatemia in 63.1% of HD patients from two dialysis centers in Syria, with only 25.3% of patients receiving P-binders other than Ca-containing binders.

Guidelines developed by the Kidney Disease Outcomes Quality Initiative (KDOQI) and the ‘Fistula First’ initiative promote the construction of AV fistulas, targeting at least 68% use in prevalent patients on dialysis^26^. In the DOPPS study from 2002 to 2003, the percentage of patients who received dialysis with an AVF ranged from 91% in Japan to 31% in the United States. All 12 countries in the study except the United States met the KDOQI recommendation of having more than 40% of patients dialyzing via AVF^27^. Over the past decade, the AVF rate in the United States for prevalent HD patients has increased from 26 to 61%. Many U.S. centers and European centers achieve much higher percentages (≥90%)^26^. It is well-known that inadequate blood flow through venous catheters remains a significant problem and usually flow is limited to a range closer to 300 ml/min. This results in a lower fractional Ur clearance (Kt/V) or as known a dialysis dose^26^. In addition, previous work from the DOPPS, Pisoni *et al*.^28^ found that patients who were dialyzing with a catheter showed a lower likelihood of having a Hb level of 11 g/dl or greater. In the same regard, patients dialyzing with a catheter were found to be greater for vascular access infection rates, the risk for infection-related hospitalization, and mortality and hospitalization compared with those using an AVF^29,30^.

Similar to these guidelines, most patients in our dialysis centers were using AVF (89.7%), and dialysis access showed a significant impact between phos groups (Table 1). Additionally, increasing time on HD showed a protective effect against hyperphosphatemia, where the odds of having hyperphosphatemia declined with increasing time on HD (OR 0.98), and the mean HD time was higher in the norm phos group compared to the high phos group. The plausible explanation is that HD access and increasing HD time have pernicious effects on dialysis dose, and on MBD such as phos levels, which reflects an increase in phos clearance.

It is well-known that eGFR is a risk factor for hyperphosphatemia^1,2,19,31^. Furthermore, hyperphosphatemia correlates with PTH and Ca, which are components of MBD^10,31^. Yet the most well-known consequences of hyperphosphatemia are its effects on parathyroid glands. High serum phos, as does low Ca, increases parathyroid cell proliferation^10^.

In our study, PTH and Ca showed a significant correlation with phos levels in addition to high mean PTH levels in both groups, which reflects a nonadherence with drug prescriptions. Other risk factors for hyperphosphatemia were presented in regression analysis. As the levels of Ur and Cr are surrogates for dialysis dose, this might explain their positive association with hyperphosphatemia, where increasing levels of Ur and Cr associated with an increasing OR for hyperphosphatemia.

A few studies mentioned the relationship between anemia and hyperphosphatemia; however, it remains a controversial issue with scant and conflicting data^18,19,25,31^. In kidney transplant patients, a linear and significant association of higher serum phos correlated with both lower Hb and with higher odds of anemia even in the subgroup analyses divided by eGFR, where every 0.8 mg/dl higher serum phos level was associated with 0.26 g/dl lower Hb concentration^18^. Higher phos levels were associated with a greater likelihood for anemia in a population with early CKD (eGFR >30 ml/min/1.73 m^2^) and normal kidney function deducing that every 0.5 mg/dl increase in phos demonstrated a 16% greater likelihood for moderate anemia (defined as Hb <11 g/dl), and serum phos levels more than 3.5 mg/dl were associated with a greater likelihood for moderate anemia^19^. Also, low Hb levels were associated with an increased risk for hyperphosphatemia (OR 1.52) among individuals without CKD^31^. One plausible explanation is that higher phos is a surrogate for other components of CKD–MBD, such as low vitamin D or increased PTH and FGF23 levels, which are linked to lower Hb levels or resistance to EPO therapy^32–36^. Another potential explanation is the effect of phos on increasing inflammation and uremic polyamines metabolism, which are uremic toxins that can inhibit erythropoiesis^37,38^.

On the other hand, the DOPPS study indicated that higher serum phos and Ca levels were found to be significantly related to a greater likelihood of having higher Hb concentration, where the adjusted OR of having Hb at least 11 g/dl equal 1.08 per 1 mg/dl phos elevation^25^. Meanwhile, DOPPS did not recommend higher ranges of phos levels, in contrast with the KDOQI guideline, in an attempt to improve anemia in HD patients because this study and other studies have shown that higher Ca and phos levels are associated with higher mortality risks, especially cardiovascular mortality^39,40^.

In our study, Hb did not show statistically significant difference between the two phos groups (divided by the high recommended level of 5.5 mg/dl). Also, Hb did not display statistically significant effect on phos levels in multivariate and univariate logistic regressions even after using forest plot. This might be by a relatively small and different study sample compared to previously mentioned studies running in a population with early CKD or normal kidney function, where the inverse relation between phos and Hb was observed^18,19,31^. By lateral, the DOPPS study, which was similar to our study participants i.e. HD patients, showed a positive correlation between phos and Hb^25^. These conflicting results mandate further studies to define the correlation between Hb and phos.

In the end, a secondary result of this study showed that the prevalence of severe anemia was 60.9%, defined as Hb levels less than 10 g/dl, which represented a poor prognosis^13,14,41^. This might be due to a poor nutrition status, which is extrapolated by low mean ALB levels (Table 2), and by insufficient dialysis dose where only two HD sessions per week are obtained in our country due to limited resources.

Limitations of the present study were a limited number of patients restricted to two dialysis centers, which may not be representative of the HD population in Syria. Also, some data that may affect anemia and hyperphosphatemia such as diet, lifestyle, ferritin, transferrin saturation, and levels of B12 and B9 were incomplete. Despite these points, we observed several comorbidities among our HD population such as anemia, hyperphosphatemia, low ALB, and elevated PTH. This mandates further studies aiming to control these comorbidities by adhering to diet and medications.

Hyperphosphatemia and anemia are well-known complications among HD patients; however, the relationship between Hb and phos is not well studied. These results mandate future studies to evaluate the correlation and further efforts to determine the range of phos that could have a benefit on anemia concerning other comorbidities like CVD.

## Conclusion

In this sample of HD patients from Syria, a high prevalence of hyperphosphatemia and anemia was observed (63.1 and 60.9%, respectively); 89.7% of patients were using AVF as dialysis access, which showed a significant impact on phos levels. Additionally, a protective effect against hyperphosphatemia was observed with increasing time on HD. The risk of hyperphosphatemia increased with higher levels of Ur and Cr. Eventually, there was no statistically significant relation between levels of phos and Hb using several statistical methods.

## Ethical approval

The study was approved in accordance with the Declaration of Helsinki and in line with the STROCSS criteria.

## Consent

Written informed consent was obtained from patients for publication of this article and any accompanying images.

## Sources of funding

None.

## Author contribution

M.A.: write and submit the manuscript, literature review, and collect data; M.K.: revised the manuscript and managed data, and literature search; M.T.A.: data analysis, write and explain the study results; B.A., R.K., A.A.S., and M.A.M.: correct the manuscript and collect data; Q.H. and K.B.: make article corrections, supervise, and follow-up.

## Conflicts of interest disclosure

There are no conflicts of interest.

## Research registration unique identifying number (UIN)

1. Name of the registry: OSF Preregistration.
2. Unique identifying number or registration ID: osf.io/z5xgw.
3. Hyperlink to your specific registration (must be publicly accessible and will be checked): https://archive.org/details/osf-registrations-z5xgw-v1

## Guarantor

Dr Mohammad Alsultan.

## Provenance and peer review

Not commissioned, externally peer-reviewed.
